# A Computer Vision Framework for Structural Analysis of Hand-Drawn Engineering Sketches

**DOI:** 10.3390/s24092923

**Published:** 2024-05-03

**Authors:** Isaac Joffe, Yuchen Qian, Mohammad Talebi-Kalaleh, Qipei Mei

**Affiliations:** 1 Department of Electrical and Computer Engineering, University of Alberta, Edmonton, AB T6G 1H9, Canada; ijoffe@ualberta.ca; 2 Department of Civil and Environmental Engineering, University of Alberta, Edmonton, AB T6G 1H9, Canada; yq12@ualberta.ca (Y.Q.); talebika@ualberta.ca (M.T.-K.)

**Keywords:** computer vision, deep learning, AI for structural engineering, structural analysis, sketch to model

## Abstract

Structural engineers are often required to draw two-dimensional engineering sketches for quick structural analysis, either by hand calculation or using analysis software. However, calculation by hand is slow and error-prone, and the manual conversion of a hand-drawn sketch into a virtual model is tedious and time-consuming. This paper presents a complete and autonomous framework for converting a hand-drawn engineering sketch into an analyzed structural model using a camera and computer vision. In this framework, a computer vision object detection stage initially extracts information about the raw features in the image of the beam diagram. Next, a computer vision number-reading model transcribes any handwritten numerals appearing in the image. Then, feature association models are applied to characterize the relationships among the detected features in order to build a comprehensive structural model. Finally, the structural model generated is analyzed using OpenSees. In the system presented, the object detection model achieves a mean average precision of 99.1%, the number-reading model achieves an accuracy of 99.0%, and the models in the feature association stage achieve accuracies ranging from 95.1% to 99.5%. Overall, the tool analyzes 45.0% of images entirely correctly and the remaining 55.0% of images partially correctly. The proposed framework holds promise for other types of structural sketches, such as trusses and frames. Moreover, it can be a valuable tool for structural engineers that is capable of improving the efficiency, safety, and sustainability of future construction projects.

## 1. Introduction

In civil engineering, a structure is a system comprising interconnected elements supporting applied loads. For the design of any functional public structure, trained and skilled structural design engineers must create and analyze mathematical models of structures to determine the extent to which these proposed systems fulfill the design objectives and satisfy safety, aesthetic, economic, and environmental considerations [1]. Comprehensive design of structures typically requires the use of complex three-dimensional models produced using commercial software, but employing two-dimensional heuristics of these complex systems expedites the initial phases of the structural design process. Generating diagrams representing the internal forces experienced by these simplified models is a crucial step in the design process. These analyses enable engineers to quickly gain an understanding of the system and provide a relatively expeditious method of evaluating the validity and effectiveness of a potential solution without the resource-intensive analysis that a more comprehensive model or structure would require. However, the process of analyzing these models by manual hand calculation is laborious and error-prone, making it a suboptimal strategy.

Alternative solutions to mitigate these disadvantages exist, but each has its own shortcomings. For instance, hand-drawn diagrams may be manually translated into a data structure consisting of nodes and elements for input into a computer program for analysis, but this process is often more time-consuming and error-prone than simply analyzing the structures by hand. As a further example, existing commercial structural analysis software [2] allows users to draft detailed structural models, but this process is just as slow and tedious as manual analysis, particularly for simple structures. Furthermore, these methods may not be user-friendly for on-site engineers, who may need to make quick decisions and take immediate actions based on the results of the structural analyses.

Despite the widespread adoption of computer-aided design (CAD) and digital structural analysis tools, what is lacking in the industry is a simple and easy-to-use tool capable of analyzing technical sketches using artificial intelligence (AI) and advanced machine-learning-based computer vision (CV) techniques directly, without the need for intermediate processing to prepare them for analysis. This paper presents a framework for a complete, rapid, and accurate workflow from a handwritten sketch to an analyzed structural model. To enhance the abstraction and convenience of the framework, all analysis must be self-contained, based only on information present in the sketch and not on any external/supplemental information from engineers. Additionally, to maximize robustness and generalizability, the tool must be able to perform offline recognition without temporal information relating to the order and speed of pen strokes. Such a tool would allow structural engineers to simply take a photograph of a rough, hand-drawn sketch and receive accurate comprehensive structural analysis results within seconds.

This paper presents a general AI-based framework to translate arbitrary handwritten engineering sketches into analyzed structural models as well as an initial implementation of a concrete system using this framework for beam diagrams. The remainder of this paper is organized as follows:

- Section 2 delves deeper into the application of AI to civil and structural engineering;
- Section 3 outlines the general framework designed and the specific system implemented;
- Section 4 presents the empirical results of the system and the accompanying analysis;
- Section 5 summarizes the conclusions of the paper and provides direction for future research.

## 2. Related Work

AI technologies have shown great advancements in recent years. While conventional computer programs map inputs to outputs by applying a series of clearly defined rules and operations outlined by a human programmer, AI technologies, especially machine learning, allow computer programs to learn the rules and operations to map inputs to outputs themselves simply by being exposed to many labeled input–output examples. The basic artificial neural network (ANN) [3], also known as multi-layer perceptron (MLP), is useful for simple classification tasks because of its natural ability to grasp patterns present in sufficiently large datasets, but it is not powerful enough to undertake difficult CV tasks. Object detection—the task of localizing and categorizing features in an image—and handwritten text recognition (HTR)—the task of recognizing and reading handwritten characters and digits in images to produce textual data—are particularly challenging. However, recent advancements in deep-learning-based AI technologies have made completing these tasks possible with ever-increasing accuracy [4,5,6]. Systems utilizing convolutional neural networks (CNNs) [7,8] have achieved state-of-the-art results across many subfields of CV [9,10]. This is because CNNs largely overcome the inherent challenges of object detection and HTR (such as the vastly different topologies from image to image and the wide variance in penmanship from person to person). Thus, most modern object detection models, including the You Only Look Once (YOLO) framework [11,12] employed in this work, and many modern HTR systems, such as the SimpleHTR architecture [13] employed herein, are implemented using CNNs. HTR systems usually apply a CNN alongside a recurrent neural network (RNN) so that sequential data can be recognized and processed with their order preserved. Simultaneous improvements to computer hardware, particularly to the graphics processing unit (GPU), have made it feasible for these large-scale deep-learning-based models to be trained and inferenced, even on consumer hardware.

In the realm of civil and structural engineering, the integration of AI technologies, particularly in CAD, structural analysis, and design, has undergone significant advancements in recent years [14,15,16,17]. Rudimentary rule-based systems capturing technical knowledge date back to the 1970s and 1980s [18,19] but are not powerful enough to solve this problem; deep-learning-based AI techniques are required. The first major application of machine-learning-based AI techniques to structural engineering came in the 1990s in the form of pattern recognition and simple design [20]. Since then, advanced AI-based systems have been applied to nearly all facets of structural engineering, from tunnel boring machines [21] to construction site safety [22]. Some recent studies have applied AI directly to the actual structural analysis process by using ANNs to predict and analyze the performance and response of particular types of structures [23,24,25,26].

CV technologies are also widely used to extract relevant information from visual data for structural analysis tasks. For instance, two-dimensional sketch-based finite element analysis (2DSketchFEA) [27] is a unified system that integrates drawing and recognition of common engineering symbols with geometric meshing, finite-element analysis, and visualization. This system aimed to provide an easy and rapid tool for analysts to study model behavioral phenomena. However, it was noted that the method did not rely on paper sketches but required users to draw on a sketchpad that recognized primitive mechanics components. Furthermore, the recognition step utilized an open-source shape recognizer rather than advanced machine-learning-based CV techniques. Sketched-truss recognition and analysis tool (STRAT) [28] was developed as a pen-based tool to expedite work in structural analysis within classrooms. It was based on classical methods of shape recognition techniques using corner-finding algorithms to locate parsing points. This tool was specifically designed to aid students with learning standard truss analysis. Similarly, sketch-based modeling and analysis of truss systems (SMATS) [29] presents a user interface that provides an iterative environment for modeling the structural behavior of sketched truss systems in real-time. This system applies gesture recognition to extract data for a structural analysis program, with results visualized in the same user interface. This work aimed to bridge the gap between architectural vision and engineering analysis, offering architects a natural environment to present and appraise structural configurations of different truss systems through sketching. Finite element analysis made easy (FEAsy) [30] is a tool that allows users to transform, simulate, and analyze their finite element models quickly and easily through freehand sketching. This tool was designed to be used in engineering education, particularly for undergraduate students in mechanical and civil engineering. It served as a learning tool for verifying answers to hand-worked problems and evaluating ideas in the preliminary stages of design projects. However, the method was based on classical least-squares optimization techniques and stroke resampling and recognition techniques. In summary, while existing studies have explored AI applications across civil and structural engineering, few have delved into utilizing deep learning and CV techniques for analyzing handwritten technical diagrams.

This paper introduces a novel framework leveraging AI and CV to recognize, understand, and analyze handwritten structural sketches. The modularized nature of the framework allows for easy extension to new features and scalability to new problems. Moreover, the framework operates autonomously, solely relying on machine-learning models to construct structural models without requiring supplemental user input.

## 3. Methodology

### 3.1. Overview

**Figure 1:**
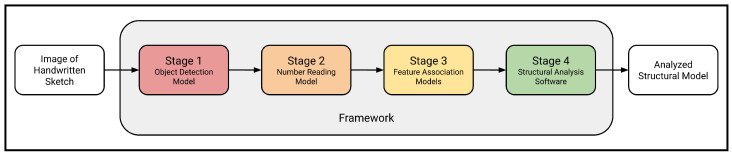
General architecture of the proposed complete framework.

Understanding and interpreting complex structural systems is a difficult and daunting task, even for professional structural engineers. In order for an AI computer program to undertake such an endeavor, the problem must be decomposed into smaller and simpler sub-problems that can each be approached and solved separately by a single machine-learning model. This multi-stage architecture follows software engineering best practices by enabling each module to be specified, designed, built, trained, optimized, tested, and maintained separately. Crucially, this approach allows for each module to be significantly altered, or even fully replaced, without affecting the rest of the system (as long as the interface between stages is kept consistent). Furthermore, this design enhances the extensibility of the system such that additional functionality may be easily integrated in the future. The problem at hand naturally lends itself to a four-stage sequence (Figure 1).

- First, in the object detection stage, all raw features in the image, including structural elements, support types, and applied loads, are classified and localized.
- Second, in the number-reading stage, all the detected handwritten numbers representing load magnitudes or element lengths in the image are read and converted into numerical data.
- Third, in the feature association stage, the relationships among the raw image features, such as whether a certain beam and support are attached or whether a given load acts on the beam, are deduced in order to construct a valid structural model that is accurately representative of the implied semantics of the sketched diagram.
- Fourth, this generated model undergoes automated structural analysis.

This divide-and-conquer approach of decomposing the recognition and analysis of structural models into distinct steps allows for loosely coupled, reusable software modules that may be easily adapted to analyze other types of structures such as trusses or frames in the future.

This work presents an initial implementation of the proposed software framework, so the system is subject to some restrictions. Certain assumptions are made concerning the format of the handwritten diagram provided, which are outlined as follows:

- Applied forces must point straight right, left, upward, or downward. Point and distributed loads drawn at an angle that is not along the *x*- or *y*-axis will be interpreted as if they were drawn only in a single direction. To circumvent this limitation, an angled load may be decomposed into two loads pointing in directions along the *x*- and *y*-axes and acting at the same point.
- Distributed forces must be uniform. Non-uniformly distributed loads behaving according to some other mathematical function will be interpreted as if they were uniform.
- Numbers must not be accompanied by units. Only digits can be read, so any letters written will produce an erroneous transcription. Existing diagrams containing numbers with units will not be analyzed properly by the system. The structural analysis software employed does not incorporate units, so any units deciphered would be discarded regardless. However, all numbers are assumed to be expressed in terms of the same system of units.
- Magnitudes must be assigned constant integer values. Variable, fractional, and decimal forces and structural proportions will be read as integers.
- Structural proportions drawn in such a manner that each length arrow begins from the same reference point must be drawn in reference to a point on the left for a horizontally oriented beam or at the top for a vertically oriented beam of the diagram.
- The diagram must be consistent. For example, structural proportions must be congruent, and an adequate number of length arrows must be supplied for the required number of elements. Any diagram that does not represent a valid structural model will produce errors.
- The diagram must be free of extraneous scribbles. Such markings will be misinterpreted as meaningful image features.

The developed software architecture is easily extensible such that resolving issues of this nature and adding new functionality are relatively straightforward.

In what follows, we describe the approach taken to construct the system that was specifically designed for structurally analyzing beam diagrams.

### 3.2. Object Detection

**Figure 2:**
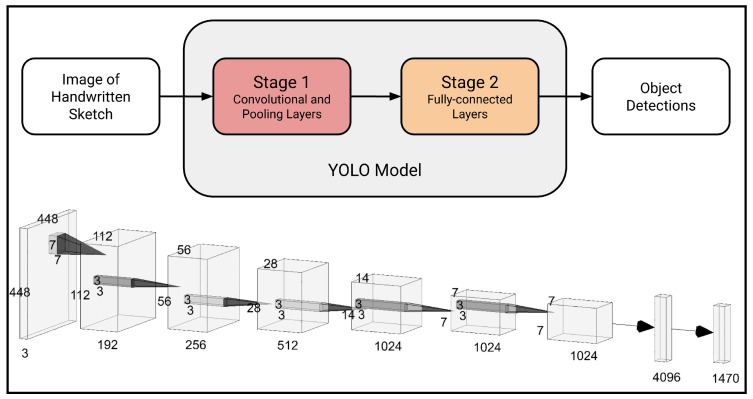
General architecture of the YOLO model [35]. Modified from [11].

The first stage of the workflow is completed using YOLOv5 [11,12], a powerful CV framework that achieves state-of-the-art results across many common CV tasks, including image classification, object detection, instance segmentation, object tracking, and human pose detection [31,32,33]. Unlike many contemporaneous alternatives, YOLO employs only a single neural network in its object detection model (Figure 2). This makes it capable of detecting and classifying objects simultaneously and also increases the training and inference speed substantially [34]. These advanced methods exhibit good performance even for images depicting complex scenes and contexts. While recognizing handwritten diagrams is simpler than many other tasks YOLO is capable of completing, it does present several key challenges that the model is able to overcome, including the wide variances in penmanship, diagram format, lighting, sketch color, and background patterns from image to image [11]. Furthermore, YOLO maintains high accuracy for overlapping and occluded features [11], which are common in hand-sketched beam diagrams. For example, loads and supports often intersect the beam, and magnitudes are often written immediately above length or load arrows.

**Figure 3:**
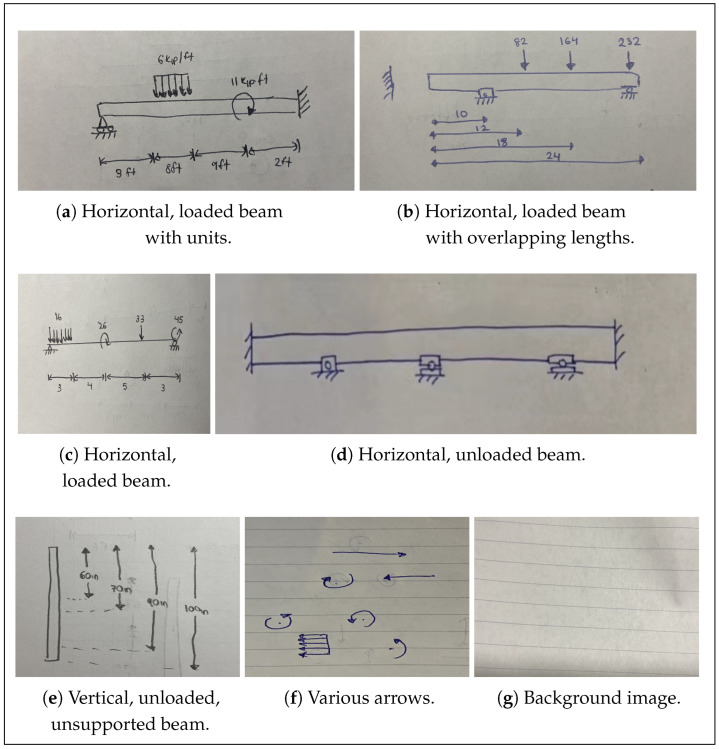
Random samples of data used for the object detection model.

To recognize the features that commonly appear in beam diagrams, the model is trained on a fully custom-made dataset of 650 images depicting hand-drawn beam diagrams and other pertinent features (Figure 3). Specifically, the model is trained to recognize beams themselves; fixed, roller, and pin support conditions; handwritten numbers; annotated dimensions of beam elements; concentrated moments applied either clockwise or counterclockwise; right-, left-, upward-, and downward-applied point forces; and right-, left-, upward-, and downward-applied uniformly distributed forces. The separation of loads pointing in different directions into different object classes allows the model to accurately determine the direction of all applied loads. Following object detection best practices, 50 background images containing no objects of interest are included in the dataset in order to reduce the number of false positives returned by the model. All 700 images in the dataset (i.e., the initial set of 650 as well as the 50 background images) are created and labeled manually during the course of the project.

Data quality is paramount for training robust machine-learned models. To minimize biases arising in the model due to limited training data sources, care is taken to ensure high dataset quality. The diagrams are meticulously drawn to encompass many different drawing styles and formats, maximizing model robustness. For example, diagrams are drawn on blank, lined, and graph paper; diagrams are sketched using blue ink, black ink, and pencil; beams are drawn both as lines and as boxes; arrows are sketched with and without a filled-in arrowhead; structural supports are drawn as simplified shapes and in detailed formats.

**Figure 4:**
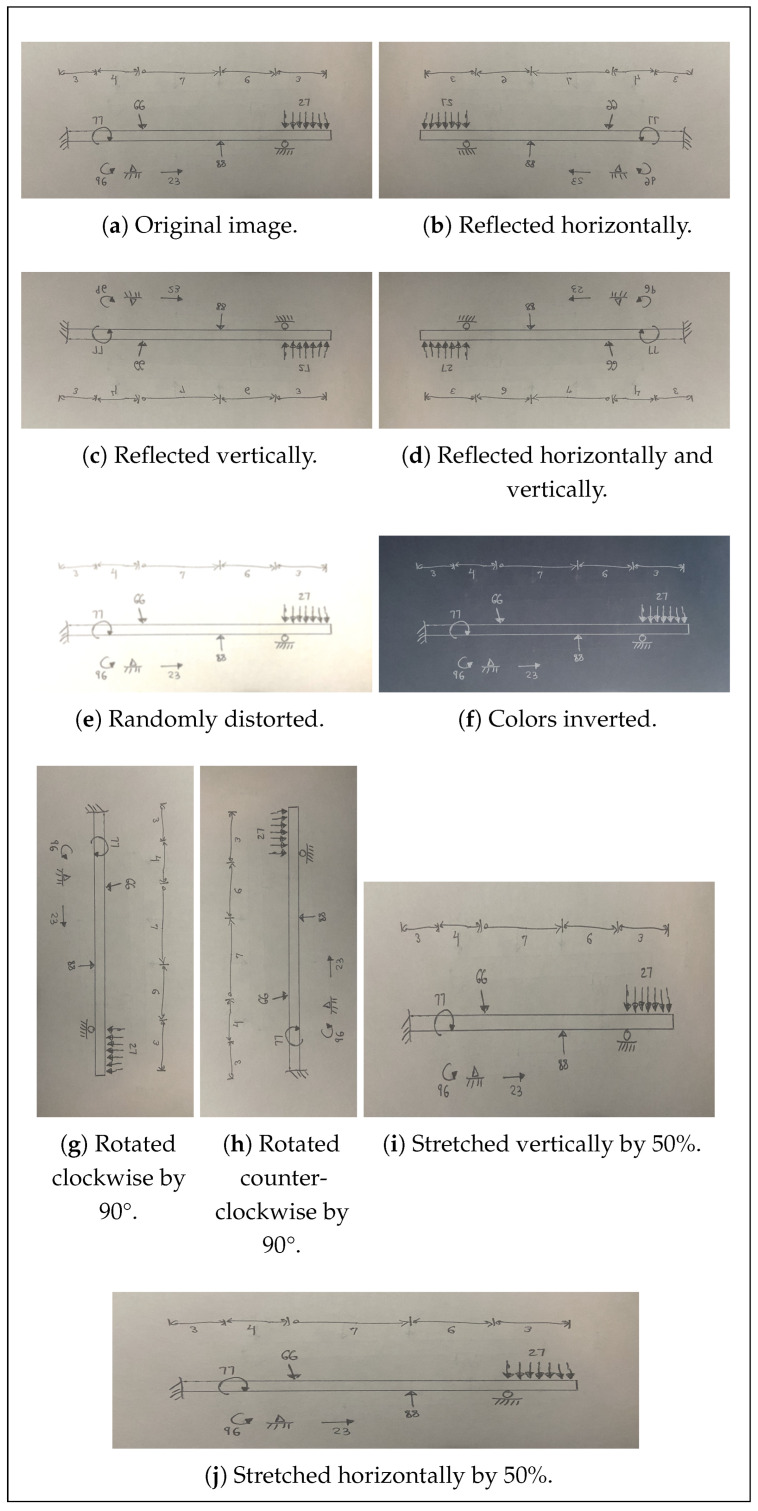
Visualization of the nine data augmentation techniques used on a random sample image.

To further minimize adverse effects from the presence of any unintended patterns present in the training dataset, each image is augmented in nine ways, producing a labeled dataset of 7000 images. In the augmentation process, each image is rotated 90° clockwise; is rotated 90° counterclockwise; is reflected horizontally; is reflected vertically; is reflected both horizontally and vertically (both augmentations at once); is stretched by 50% horizontally; is stretched by 50% vertically; is subject to random distortions, such as blurring, brightness changes, and contrast changes; and is subject to color inversion (Figure 4). Image rotations and reflections serve to eliminate many of the unintended topological patterns that can appear by virtue of the manner in which diagrams tend to be drawn, making the model more generalizable to different engineers’ diagrams. These types of alterations also balance the counts of each direction of loads in the dataset, since reflections produce a counterpart pointing in each direction for each load drawn. Stretching and distorting the images, meanwhile, exposes the model to diagrams mimicking those sketched by different people. Color inversion, moreover, produces a light-colored diagram sketched on a black background, enabling the model to support the digitally generated diagrams commonly produced on tablets. Importantly, this augmentation procedure creates a sufficiently large dataset—7000 images—to properly train the model (Table 1). While it is recommended that YOLO training datasets contain as many as 1000 images and 10,000 instances per class for detection [12], the simple nature of the problem at hand allows for strong performance even with a smaller dataset. A small dataset for model validation containing 30 analogous, but separate, images is also created. Applying the same augmentation procedure results in a dataset containing 300 images, representing a 4.1%/95.9% validation–training split.

### 3.3. Number Reading

**Figure 5:**
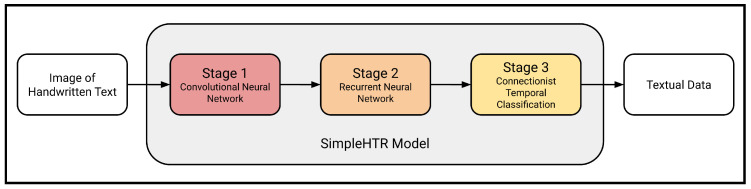
General architecture of the SimpleHTR model. Modified from [13].

The second stage of the system is completed by SimpleHTR [13], which is a separate number-reading CV model applied to all the numbers detected in the previous stage. The YOLO model accurately detects and segments numbers and produces a minimal bounding box for each handwritten number in the image. The coordinates of this box are used to crop the input photograph, producing an image containing only the relevant number to be read. Reading single handwritten digits is already challenging due to the variations in the appearance of handwriting from one individual to another, but transcribing a sequence of digits is an even more difficult task. Handwritten numbers often, but not always, contain overlapping digits that are nearly impossible to segment, and the number of digits to detect is unknown prior to execution. The architecture underlying the model consists of a CNN followed by an RNN (Figure 5); the CNN allows patterns representing the digits to be detected anywhere in the input image, and the RNN allows many digits to be detected in succession.

To enable it to read variable-length handwritten numbers written by different people, the model is trained on a large synthetic dataset designed to represent real-world examples. The custom-made training dataset consists of 600,000 images based on the comprehensive data available in the Modified National Institute of Standards and Technology (MNIST) dataset [36]. The MNIST dataset contains 60,000 images for training and 10,000 images for testing. This dataset consists of high-contrast, grayscale, 28 × 28 pixel images of single handwritten digits written by approximately 250 high school students and United States government employees. This large, high-quality dataset is closely representative of real-world handwritten numbers, making it an ideal data source for training and benchmarking HTR models; models trained on it tend to be generalizable to real-world data.

**Figure 6:**
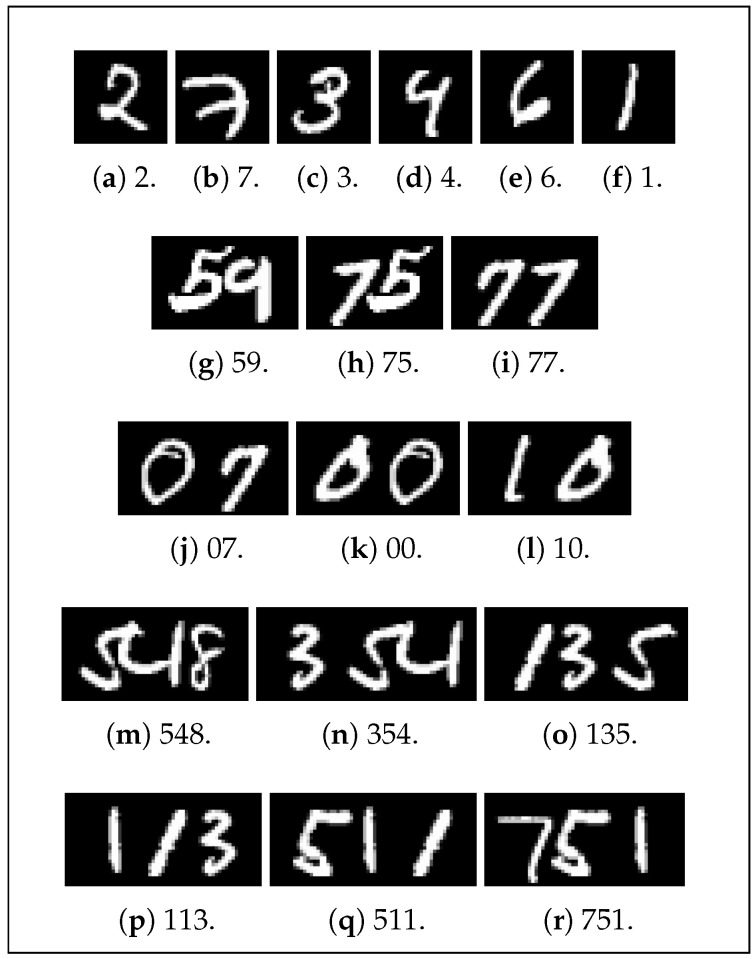
Random samples of data used for the number-reading model, with the correct number to be read.

Significant MNIST dataset processing is required to allow transcription of multi-digit numbers. To ensure single digits can still be read, all 60,000 original training images are incorporated into the custom-made dataset as is. To generate plausible two-digit numbers, each original image is concatenated with the next image in the dataset with a random amount of overlap. This overlap is intended to represent various styles and densities of handwriting (i.e., varying degrees of proximity between neighboring digits). This process is then repeated, but in reverse, meaning that each pair of numbers is concatenated in the opposite order. As a result, 120,000 images of random two-digit numbers are produced. Three-digit numbers are generated in a similar manner—each image is randomly concatenated with the next two and the previous two images in the dataset. These 120,000 three-digit number images, together with the 120,000 two-digit numbers and the 60,000 original images, make up the initial 300,000-image dataset (Figure 6).

**Figure 7:**
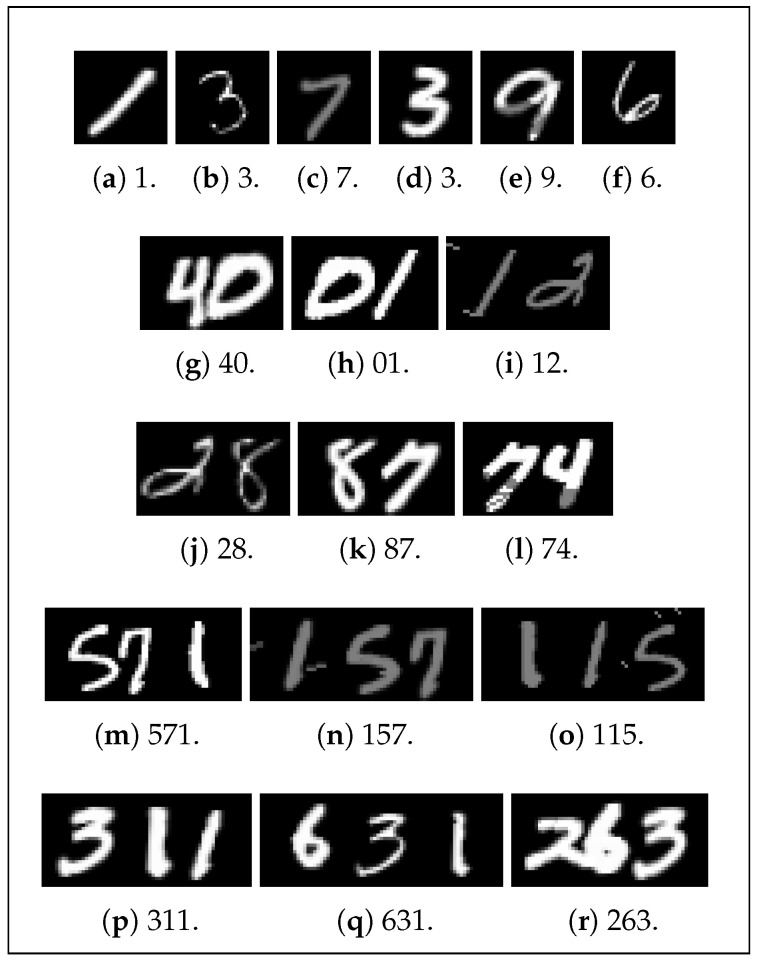
Random samples of augmented data used for the number-reading model, with the correct number to be read.

Following the construction of this MNIST-based dataset, each image is randomly altered in various ways to produce an augmented copy of itself, bringing the total to 600,000 training images (Table 2). Specifically, each image is subject to random text bolding or thinning; random addition of both dark and light noise; and random small rotations, shifts, and stretches (Figure 7). This augmentation is intended to introduce the kind of data that may be encountered in real-world applications, where text thickness varies, random noise is common, and images are not always level. The randomness involved in the MNIST dataset and the dataset production process creates a diverse dataset, increasing the generalizability of the model to different styles of handwriting. On the other hand, this augmentation also results in some low-quality images being generated. For example, certain digits may overlap too much, preventing even humans from correctly reading the number, while others may not overlap enough, creating unnatural gaps between digits that likewise make reading difficult. With a dataset this large, it is not feasible to thoroughly manually inspect data quality, but random sampling indicates that the majority of images are plausible. While this training dataset only includes one-, two-, and three-digit numbers, the inherent abstraction of the RNN enables the model to read longer numbers that may be encountered. A smaller dataset for model validation containing 30,000 analogous but separate images is also created. This dataset is constructed in the same fashion based on the 10,000 testing images in the MNIST database, except that the numbers are not concatenated in reverse-order and no augmentations are applied. This dataset represents a 4.8%/95.2% validation–training split.

### 3.4. Feature Association

The third stage of the system is completed by a series of custom-made MLPs [37]. These data-driven classifiers are used to ascertain the relationships among image features as part of the algorithm in order to create a structural model. It should be noted that deep learning allows for excellent performance on classification- and recognition-style tasks, even on highly unstructured datasets (such as the one at hand, where the topology and scale of each diagram varies greatly). The MLP model determines the rules for classification itself, without being directly influenced by human biases, and its performance can be improved simply by expanding the training dataset.

To analyze the various relationships among image features, six custom MLPs are designed and implemented using TensorFlow [38]. These ANNs are used to determine (1) whether a given beam and support are connected (“beam–support”), (2) whether a given applied load acts on a beam (“beam–load”), (3) whether a given handwritten number corresponds to some applied load (“load–number”), (4) whether a given length arrow is associated with some element in the structural model representing a beam (“element–length”), (5) whether a given handwritten number corresponds to some length arrow (“length–number”), and (6) whether the length arrows in the image are written in an overlapping or in a separated format (“length–style”). All datasets for these models are obtained by manually labeling all the relationships in the previously described 730-image dataset of beam diagrams, maintaining the initial training–validation split.

**Figure 8:**
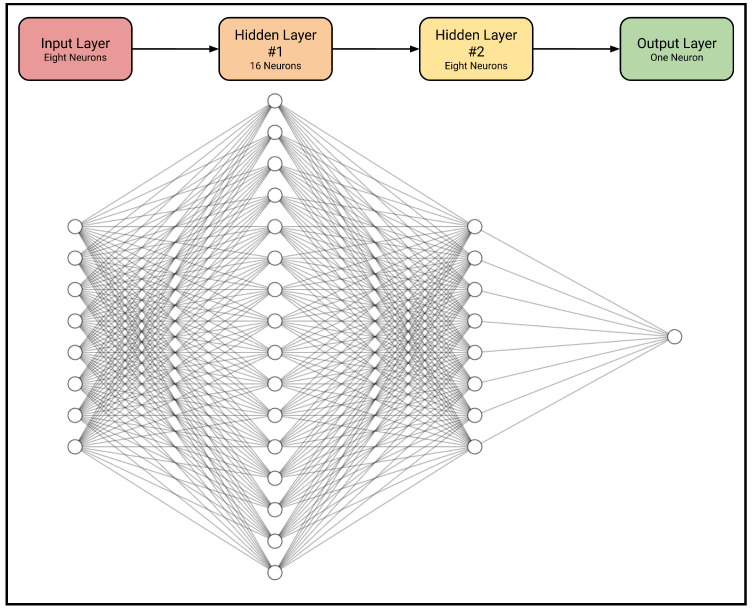
General architecture of the beam–support, beam–load, load–number, element–length, and length–number MLPs [35].

The first five MLPs map eight input variables representing the four corner coordinates of the bounding boxes of the relevant objects to one output variable. This output is a numerical value between 0 and 1 representing the likelihood that the relevant condition is true. To undertake this task, the model uses two hidden intermediate layers consisting of 16 and 8 neurons, respectively (Figure 8). Thus, these models have 289 parameters. Conversely, the “length–style” MLP takes 20 inputs corresponding to the four bounding coordinates of any five lengths present in the image in any order, but it still produces one analogous output indicating whether the lengths are overlapping or separated. This MLP employs two hidden layers with 32 and 8 neurons, respectively, making it a 945-parameter model.

To ensure all the MLPs are independent of the absolute image size, all coordinates inputted are normalized to a fraction of the largest coordinate present along the given axis. Each training example is subject to three different augmentations in order to minimize bias within the training datasets. These augmentations are to reflect each input horizontally, reflect each input vertically, and reflect each input both horizontally and vertically simultaneously. Moreover, to ensure that the length–style MLP is independent of the order in which lengths are inputted into the model, each training example is reordered in every possible permutation, even for those images with more than five lengths. While these datasets are produced using only the 730-image dataset described above, they are large enough to train the MLPs at hand. In general, the number of training examples in a dataset should be at least 10 times the number of parameters in the model to be used [39]. Thus, for the present case, the 289-parameter MLPs should be trained on at least 2890 examples, and the 945-parameter MLP should be trained on at least 9450 examples. All six datasets created far surpass this benchmark (Table 3), indicating that the MLPs can be properly trained on this data. Furthermore, these datasets contain large splits for validation and thus are well suited to meaningfully evaluate the MLPs; the validation–training split for the individual datasets ranges from 5.8–94.2% to 19.1–80.9%, and the average split is 12.5–87.5%.

**Figure 9:**
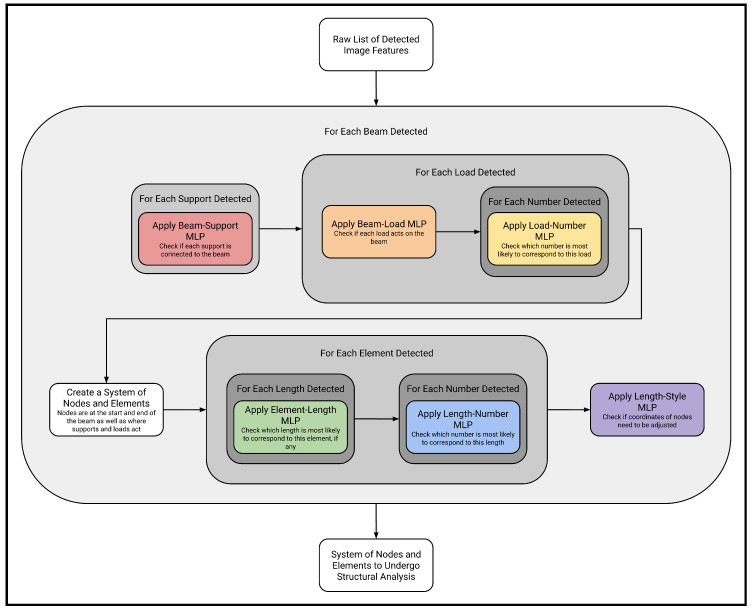
General architecture of the algorithm to convert features into a structural model.

These MLPs are applied in a structured algorithm to convert the raw data extracted by the previous CV models into a format that can be further analyzed. More precisely, the goal of the algorithm is to translate a list of raw information about detected objects into a coherent beam data structure that encompasses all relevant information about the given beam system and that that can be easily incorporated into structural analysis software. Each structural model must contain exactly one beam, so each beam detected is considered separately by the algorithm. The step-by-step execution of the algorithm (Figure 9) is described below.

First, each support detected in the image must be tested to determine whether there is a relationship to the beam at hand. The coordinates of the bounding boxes of the beam and support being considered are passed into the beam–support MLP, and if the corresponding output exceeds the defined threshold value, the beam and support are determined to be related. In this context, it should be noted that a “relationship” means that the support exerts reaction forces on the beam. If the given beam and support are deemed to be related, relevant information about the support is incorporated into the beam’s data structure.

Second, in a similar fashion, each applied load detected in the image must be tested to determine whether there is a relationship to the beam at hand. The coordinates of the bounding boxes of the beam and load being considered are passed into the beam–load MLP, and if the corresponding output exceeds the defined threshold value, the beam and support are deemed to be related. Here, a “relationship” means that the load acts on the beam. If the given load and beam are deemed to be related, relevant information about the applied load is incorporated into the beam’s data structure. However, unlike in the case of supports, further investigation is required at this juncture: if a load is determined to be acting on the beam, its associated magnitude must be ascertained.

Third, each handwritten number detected in the image is tested to determine whether there is a relationship to the load that was just discovered to be acting on the beam. The coordinates of the bounding boxes of the load and number being considered are passed into the load–number MLP, and whichever number produces the highest output value is deemed to be related to the load. In this case, a “relationship” means that the number represents the magnitude of the load. The numerical value represented by this handwritten number—this number having been read earlier—is incorporated into the information about the load at hand in the beam’s data structure. How this magnitude is interpreted depends on the given type of load: a point load, a distributed load, or a couple moment. The beam data structure resulting from the completion of this step of the algorithm contains information about the beam as well as its supports and loads, including where these objects are located along the beam. At this juncture, the beam system can be interpreted as a one-dimensional series of elements connecting structural nodes. Structural nodes occur at the start and end of the beam as well as at each occurrence of a support or load. At this point in the process, the location of each node along the beam is assumed to be at an edge of the beam bounding box or at the center of the bounding box of the support or load acting at the node.

Fourth, each element contained in this rudimentary data structure and each length arrow detected in the image must be tested to determine whether a relationship exists. The coordinates of the structural element and of the bounding boxes of the length being considered are passed into the element–length MLP, and whichever length produces the highest output value that also exceeds the defined threshold value is deemed to be related to the element. In this case, a “relationship” means that the element’s length is given by the length arrow. If no length value exceeds the minimum threshold for a particular element, that element is discarded, as the nodes joined by the element are one and the same. The data structure is compressed accordingly, allowing loads and supports to act at the same node despite their bounding boxes having slightly different center locations. Just as in the case of loads, though, further investigation is required at this juncture; if a given length is determined to be associated with a given structural element, its associated magnitude must be ascertained.

Fifth, each handwritten number detected in the image must be tested to determine whether there is a relationship to the length that was just discovered to be associated with a structural element. The coordinates of the bounding boxes of the length and number being considered are passed into the length–number MLP, and whichever number produces the highest output value is deemed to be related to the length. Similar to the load–number case, a “relationship” means that the number represents the magnitude of the length. The numerical value represented by this handwritten number—this number having been read earlier—is incorporated into the information about the location of the nodes in the beam’s data structure.

Sixth, the style of length arrows drawn must be tested. As many lengths as possible are passed into the length–style MLP, and if the corresponding output exceeds the defined threshold value, the lengths are deemed to have been drawn separately. This means that each length arrow is drawn relative to the previous one and not to a common point. In this case, all coordinates of nodes must be adjusted to account for the length of the previous elements. This produces a coherent data structure consisting of a series of nodes, each with corresponding supports and loads.

### 3.5. Structural Analysis

The fourth stage in the system is completed using OpenSees structural analysis software [40]. The same algorithm employed in the previous step converts the image features into a comprehensive beam data structure taking the form of a collection of nodes and edges. These are the structural nodes upon which loads and supports act and the structural elements of varying lengths that join them. These data are incorporated into OpenSees (OpenSees nodes are instantiated, positioned, fixed, and loaded appropriately for each beam node, and OpenSees elements are instantiated to join these nodes), producing an analyzable structural model. At this juncture, any desired structural analysis and visualization supported by OpenSees is carried out. Specifically, the model structure, loads, and deformation are visualized, and the beam’s axial force, shear force, and bending moment diagrams are plotted. This structural analysis provides useful information to structural engineers concerning the efficiency and viability of potential designs in an easy-to-understand format.

## 4. Results

### 4.1. Object Detection

**Figure 10:**
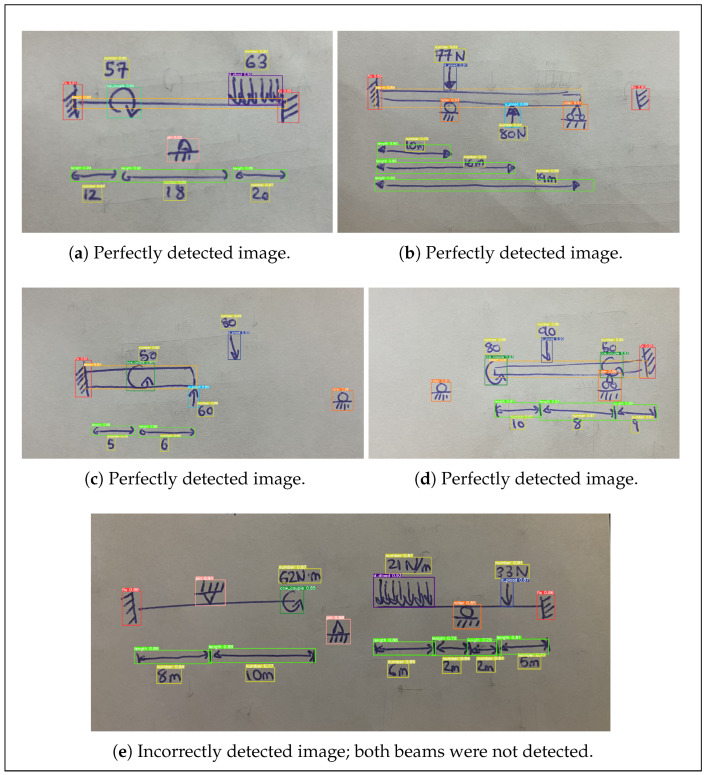
Selected samples of outputs generated by the YOLO object detection model.

The YOLOv5s model architecture, which offers good model performance, low computational burden, and high speed, was selected for the system. A YOLOv5s model starting with pre-trained model weights was trained for 35 epochs on the associated dataset of 7000 labeled images. When evaluated on the separate 300-image validation dataset using a confidence threshold of 85.0% and an intersection-over-union threshold of 50.0%, the model achieved a total precision of 98.6%, a total recall of 96.1%, and a total mean average precision of 97.8% (Table 4). Although the model experienced some challenges in detecting beams themselves and in detecting overlapping length arrows and numbers (Figure 10), with just one other instance of error, all other objects were classified entirely correctly among the non-augmented images in the validation dataset.

### 4.2. Number Reading

Three CNN layers were added to the baseline number-reading model architecture (increasing the number of CNN layers to eight) alongside the original two RNN layers. The model was trained from scratch for 20 epochs, resulting in a digit error rate of 0.5% and an overall number accuracy of 99.0% on the separate 30,000-image validation dataset (Table 5). Intuitively, the character error rate was found to be largely independent of the length of the number being read. Although there was slightly better performance on isolated digits compared to digits contained within a larger string, this was presumably attributable to the excessively overlapping digits present in some of the multi-digit examples. Given that all digits must be read perfectly in order for a number to be read correctly, it stands to reason that longer numbers tend to have a slightly lower accuracy. However, since both the training and validation datasets were generated by the same automated process and did not contain any naturally written multi-digit numbers, the generalizability of the model to real-world data needs to be further explored. The extensive data augmentation procedure and the expansive nature of the original MNIST dataset work to mitigate these issues, but the model’s real-world performance cannot be tested on a large scale at present due to the lack of data.

### 4.3. Feature Association

The MLPs were designed, trained, and tested using TensorFlow [38]. The beam–support, beam–load, load–number, element–length, and length–number MLPs were each trained for 50 epochs, while the larger length–style MLP was trained for 30 epochs. These models achieved accuracies ranging from 95.1% to 99.5% on the separate validation datasets using a confidence threshold of 0.5 for binary classification (Table 6). Overall, the average accuracy of these perceptrons was found to be 98.2% on the separate validation datasets, indicating strong performance and no overfitting to the training data. Despite the elementary machine-learning techniques utilized, these accurate results can be considered plausible.

In practice, the load–number, element–length, and length–number MLPs are used only within the context of the feature conversion algorithm. This allows them to achieve better performance, since this algorithm compares numerical probabilities and not classifications. For example, if a labeled positive pair produces a model output less than the threshold but greater than all other relevant pairs, it is interpreted as incorrect according to binary classification but is actually correct in the context of the broader algorithm.

### 4.4. Structural Analysis

This stage of the system is the only one that does not involve machine learning—it merely applies static structural analysis rules. As such, there is no associated accuracy for this stage. In other words, if the previous stages have generated a structural model that accurately represents the handwritten diagram, the analysis produced by this stage will be correct.

### 4.5. Overall System

To meaningfully analyze the end-to-end performance of the system built, a 20-image testing dataset (separate from the larger and more comprehensive validation datasets for each model) was crafted to cover a diverse range of valid diagrams. For example, diagrams were drawn on blank, lined, and graph paper; diagrams were sketched using blue ink, black ink, and pencil; beams were drawn both as lines and as boxes; arrows were sketched with and without a filled-in arrowhead; structural supports were drawn in both simplified and detailed formats. The system produced entirely correct structural analyses for nine images in the dataset, representing an accuracy of 45.0% (Table 7). The rate at which individual beam systems present in the images were analyzed correctly was slightly higher at 47.8% (11 of the 23 beam systems).

**Figure 11:**
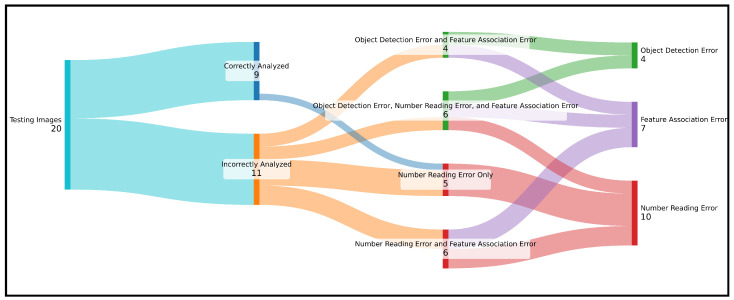
Visualization of results of complete model on testing dataset. Note that one number was read incorrectly in a correctly analyzed image, but that number represented the magnitude of a load that was not acting on the beam.

**Figure 12:**
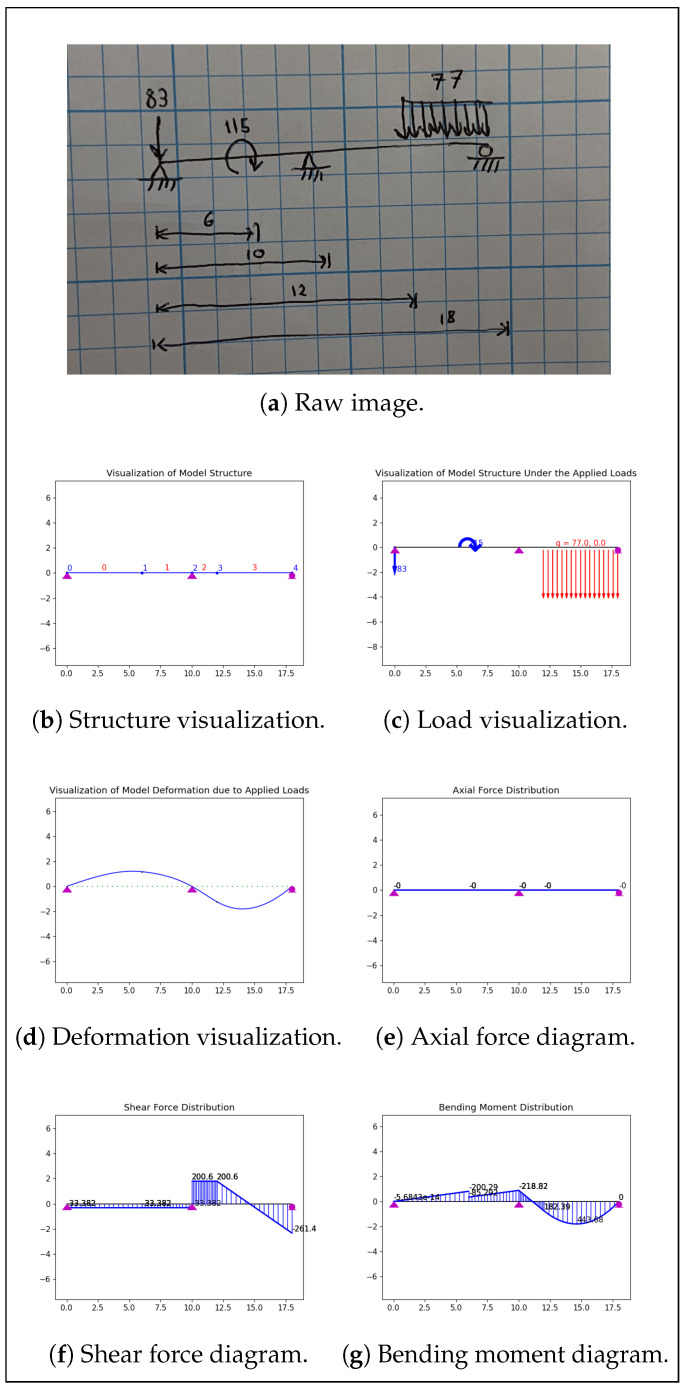
Selected sample of outputs generated by the complete system.

Manual inspection of the system results at each stage in image processing provides further insights into the strengths and weaknesses of the given application. In the present case, the YOLO object detection model performed perfectly for 80.0% of the images. More specifically, of the 329 features present across the entire dataset, the model correctly detected 324 while only falsely detecting 10; this corresponds to a precision of 97.0% and a recall of 98.5%. The number-reading model, meanwhile, transcribed all numbers perfectly for just 50.0% of images, making this the weakest link in the workflow. For this model, 111 of the 122 numbers were read correctly, resulting in an accuracy of 91.0%. This was lower than the accuracy achieved on the validation dataset, but this is to be expected since these were real-world data (i.e., not MNIST-based). The feature association models worked together to convert the detected features into the desired structural models for 65% of images. The only errors encountered in this stage were some instances of erroneously connecting supports and improperly mapping length arrows onto structural elements, and these errors typically coincided with object detection issues. In this regard, it is common for errors arising in a given stage to occur simultaneously (Figure 11). On the other hand, when all system models perform correctly, correct structural analyses are produced (Figure 12).

There are inherent challenges to achieving high accuracy in the multi-stage, machine-learned approach taken. In addition to each individual stage achieving high accuracy for its respective task, all stages need to function correctly at once in order to produce an overall correct result. Recognizing and analyzing a typical beam diagram usually involves around 100 applications of a machine-learned model. Even if each model applied were 99.0% accurate, the likelihood of all 100 inferences being correct would be just 36.6%. In the context of the case at hand, if even one error is made by any of the models in any of the first three stages—for example, a support is not identified or a number is misread—the entire structural analysis will be incorrect.

However, determining whether or not the result produced by the system is correct is straightforward since the system tends to fail in different places than a human would. For instance, the most common source of error in the entire workflow is misreading a number, but in such cases, a quick inspection of the structure and load visualizations generated will reveal the incorrect dimension or load magnitude. Humans almost never incorrectly read numbers, so this error would be easily caught by an attentive engineer. Similarly, other common errors, such as incorrectly connecting a structural support to the beam or incorrectly mapping the sketched lengths onto the right beam elements, can be easily identified in the structural visualizations produced. Again, humans would be unlikely to misinterpret the connections represented by the diagram or where length arrows project. Conversely, even trained engineers commit arithmetical errors in hand calculations, whereas when provided with a coherent structural model, the structural analysis software will not commit errors. This means that if no obvious issues can be identified in the visualizations created, the rest of the output is likely correct. Furthermore, if the analysis undertaken by the system is identical to that undertaken by an engineer, both are likely correct.

The system’s functional accuracy and predictable performance underscore its potential utility in practice. While no direct comparison can be made between the system’s performance and that of trained structural engineers because of the lack of such an existing dataset or metric, it stands to reason that the system would perform more poorly in terms of raw accuracy. However, the system still has utility since it is intended merely as an assistive tool for engineers. When applied by an attentive engineer, simple system errors can be easily rectified and acceptable results can be achieved. The most notable benefit of this system is the potential time savings; by freeing engineers from the need to perform tedious calculations, the efficiency of the overall design process may be improved. A formal and rigorous speed comparison is outside the scope of this paper; however, the presented system typically analyzed each of the 20 images in the testing dataset in about 30 s, while one of the authors, a trained structural engineer, took an average of 7 min and 43 s to manually analyze each sketch. Because of the limitation that the system requires a degree of legibility and diagram simplicity, it is not a replacement for skilled structural engineers.

## 5. Conclusions and Future Work

Overall, the system developed achieves encouraging results, and the framework designed holds promise for other hand-drawn sketch recognition and analysis problems. With future optimization and implementation of continually emerging AI technologies, the results of systems implementing this versatile framework will only continue to improve. The flexible and extensible nature of the framework presented makes it easily scalable to new data sources and adaptable to new application domains. The system shows the potential to be a valuable tool to assist engineers with quick structural analysis, expediting their design iterations and streamlining the overall workflow. It can be expected to be particularly useful for on-site engineers, who rely heavily on rough, handwritten sketches to make rapid choices. The tool may also have utility in educating new civil and structural engineers by helping them to better understand the overall structural analysis process. Because the system generally fails in different situations than humans do, it also provides students with a useful method to quickly verify their own analyses on arbitrary practice problems.

Further improvements to this system can be made in several respects. Obtaining large and diverse datasets containing diagrams sketched by many different people will improve system robustness, making the models utilized more generalizable to diverse and low-quality inputs. Additionally, the system may be extended to overcome its current shortcomings as detailed above. Notable functionalities to be investigated in future work include the capability for applied loads to point in any arbitrary direction; the capability of distributed loads to be non-uniform (i.e., following some non-constant mathematical function); the capability for handwritten numbers to be accompanied by their units; the capability of applied loads and structural proportions to hold decimal, fractional, and even variable magnitudes; and the capability for extraneous scribble marks to be ignored. Other extensions to current system functionality may also be considered, such as the capability to manually alter the structural models generated to quickly resolve small errors. Moreover, given the modularity of the design, improved versions of each individual component of the framework can be incorporated in future work as they emerge.

Future work may also involve extending the proposed framework to other structural systems, such as truss and frame structures. In this regard, the modular nature of the framework will make creating these new systems straightforward. The proposed design also allows for certain software modules in the system, including the number-reading model and certain MLPs (e.g., the load–number and length–number models) to be reused. Moreover, it allows for other software modules, such as the object detection model and certain MLPs, including the beam–support and the element–length models, to be effortlessly adapted to analogous tasks by simply adding new training data. Additionally, this general framework has the potential for interdisciplinary applications to other engineering problems. By simply obtaining the requisite training data and retraining each machine-learned model, this framework can be applied to new domains. For example, systems may be developed to recognize and analyze hand-sketched free body diagrams in mechanical engineering or to recognize and analyze handwritten circuit diagrams in electrical engineering.

## Figures and Tables

**Table 1:** Quantitative information about the dataset used for the object detection model.

Object Class		Training Count	Validation Count
Element	Beam	6040	320
Support	Fix	4830	380
	Pin	4330	250
	Roller	3640	220
Point Force	Right	1067	55
	Left	995	51
	Up	976	77
	Down	1452	137
Distributed Force	Right	442	36
	Left	442	36
	Up	372	38
	Down	644	70
Couple Moment	Clockwise	1888	114
	Counterclockwise	2182	126
Other	Length	5770	920
	Number	12,110	1660

**Table 2:** Quantitative information about the dataset used for the number-reading model.

Type of Number	Training Count	Validation Count
Single-Digit	120,000	10,000
Two-Digit	240,000	10,000
Three-Digit	240,000	10,000
All	600,000	30,000

**Table 3:** Quantitative information about the dataset used for the feature association models.

Type of Model	Training Count	Positive Share	Validation Count
Beam–Support	6064	72.8%	376
Beam–Load	3288	71.0%	320
Load–Number	12,348	21.1%	1724
Element–Length	7736	19.5%	1828
Length–Number	11,500	19.3%	2204
Length–Style	15,496	69.4%	2244
Average	9405	-	1449

**Table 4:** Results of trained YOLO object detection model on the validation dataset.

Object Class		Validation Count	Precision	Recall	mAP
Element	Beam	320	94.6%	88.4%	93.2%
Support	Fix	380	100.0%	100.0%	99.5%
	Pin	250	98.8%	97.2%	98.5%
	Roller	220	94.8%	99.1%	99.2%
Point Force	Right	55	100.0%	94.5%	97.3%
	Left	51	100.0%	88.2%	94.1%
	Up	77	100.0%	90.9%	95.5%
	Down	137	100.0%	99.3%	99.5%
Distributed Force	Right	36	100.0%	100.0%	99.5%
	Left	36	100.0%	100.0%	99.5%
	Up	38	100.0%	100.0%	99.5%
	Down	70	100.0%	100.0%	99.5%
Couple Moment	Clockwise	114	97.3%	96.5%	97.8%
	Counterclockwise	126	95.3%	96.8%	98.2%
Other	Length	920	97.8%	93.9%	96.8%
	Number	1660	99.7%	93.0%	96.4%
All	All	4490	98.6%	96.1%	97.8%

**Table 5:** Results of trained number-reading model on the validation dataset.

Type of Number	Validation Count	Character Error Rate	Word Accuracy
Single-Digit	10,000	0.4%	99.6%
Two-Digit	10,000	0.5%	99.0%
Three-Digit	10,000	0.5%	98.5%
All	30,000	0.5%	99.0%

**Table 6:** Results of trained MLPs on their respective validation datasets.

Type of Model	Validation Count	Precision	Recall	Accuracy
Beam–Support	376	98.3%	100.0%	98.9%
Beam–Load	320	99.2%	99.2%	98.8%
Load–Number	1724	98.0%	99.3%	99.5%
Element–Length	1828	96.4%	91.0%	97.6%
Length–Number	2204	95.8%	98.4%	99.0%
Length–Style	2244	99.1%	93.3%	95.1%
Average	1449	97.8%	96.9%	98.2%

**Table 7:** Results of complete system on testing dataset.

Model Stage	Individual Testing Count	Individual Accuracy	Image Accuracy
Object Detection	329	P: 97.0%, R: 98.5%	85.0%
Number Reading	122	91.0%	50.0%
Feature Association	-	-	65.0%
Structural Analysis	-	-	-
Overall	23	47.8%	45.0%

## Data Availability

Some data, models, and code that support the findings of this study are openly available in GitHub at https://github.com/mqp2259/CV4BeamAnalysis, accessed on 29 April 2024.

## References

[1] Hibbeler R. (2017). Structural Analysis.

[2] (2023). SAP2000.

[3] Rosenblatt F. (1957). The Perceptron, a Perceiving and Recognizing Automaton Project Para.

[4] Zhao Z.Q., Zheng P., Xu S.T., Wu X. (2019). Object Detection with Deep Learning: A Review. IEEE Trans. Neural Netw. Learn. Syst..

[5] Xiao Y., Tian Z., Yu J., Zhang Y., Liu S., Du S., Lan X. (2020). A review of object detection based on deep learning. Multimed. Tools Appl..

[6] Wang Y., Xiao W., Li S. (2021). Offline Handwritten Text Recognition Using Deep Learning: A Review. J. Phys. Conf. Ser..

[7] Lecun Y., Bottou L., Bengio Y., Haffner P. (1998). Gradient-based learning applied to document recognition. Proc. IEEE.

[8] Lecun Y., Boser B., Denker J., Henderson D., Howard R., Hubbard W., Jackel L., Touretzky D. (1990). Handwritten digit recognition with a back-propagation network. Proceedings of the Advances in Neural Information Processing Systems (NIPS 1989).

[9] Gu J., Wang Z., Kuen J., Ma L., Shahroudy A., Shuai B., Liu T., Wang X., Wang G., Cai J. (2017). Recent Advances in Convolutional Neural Networks. arXiv.

[10] Li Z., Liu F., Yang W., Peng S., Zhou J. (2022). A Survey of Convolutional Neural Networks: Analysis, Applications, and Prospects. IEEE Trans. Neural Netw. Learn. Syst..

[11] Redmon J., Divvala S., Girshick R., Farhadi A. (2016). You Only Look Once: Unified, Real-Time Object Detection. arXiv.

[12] Jocher G. (2020). YOLOv5 by Ultralytics, 7.0.

[13] Scheidl H. (2018). Handwritten Text Recognition in Historical Documents. Diploma’s Thesis.

[14] Tapeh A.T.G., Naser M.Z. (2023). Artificial Intelligence, Machine Learning, and Deep Learning in Structural Engineering: A Scientometrics Review of Trends and Best Practices. Arch. Comput. Methods Eng..

[15] Málaga-Chuquitaype C. (2022). Machine Learning in Structural Design: An Opinionated Review. Front. Built Environ..

[16] Salehi H., Burgueño R. (2018). Emerging artificial intelligence methods in structural engineering. Eng. Struct..

[17] Baduge S., Thilakarathna S., Perera J., Arashpour M., Sharafi P., Teodosio B., Shringi A., Mendis P. (2022). Artificial intelligence and smart vision for building and construction 4.0: Machine and deep learning methods and applications. Autom. Constr..

[18] Bennett J., Creary L., Englemore R., Melosh R. (1978). SACON: A Knowledge-Based Consultant for Structural Analysis.

[19] Maher M.L. (1987). Expert Systems for Structural Design. J. Comput. Civ. Eng..

[20] Vanluchene R.D., SUN R. (1990). Neural Networks in Structural Engineering. Comput.-Aided Civ. Infrastruct. Eng..

[21] Li X., Zhao S., Shen Y., Xue Y., Li T., Zhu H. (2024). Big data-driven TBM tunnel intelligent construction system with automated-compliance-checking (ACC) optimization. Expert Syst. Appl..

[22] Sulaiman S., Zulaiha P., Ali M.I., Ramli N.I., Jamaludin O., Shu Ing D. (2023). How Artificial Intelligence Changed the Construction Industry in Safety Issues. IOP Conference Series: Earth and Environmental Science.

[23] Kiani J., Camp C., Pezeshk S. (2019). On the application of machine learning techniques to derive seismic fragility curves. Comput. Struct..

[24] Sivandi-Pour A., Farsangi E., Takewaki I. (2020). Estimation of Vibration Frequency of Structural Floors Using Combined Artificial Intelligence and Finite Element Simulation. J. Eng. Res..

[25] Mangalathu S., Jeon J.S. (2018). Classification of failure mode and prediction of shear strength for reinforced concrete beam-column joints using machine learning techniques. Eng. Struct..

[26] Mangalathu S., Jang H., Hwang S.H., Jeon J.S. (2020). Data-driven machine-learning-based seismic failure mode identification of reinforced concrete shear walls. Eng. Struct..

[27] Hutchinson T.C., Kuester F., Phair M.E. (2007). Sketching finite-element models within a unified two-dimensional framework. J. Comput. Civ. Eng..

[28] Peschel J.M., Hammond T.A. STRAT: A Sketched-truss Recognition and Analysis Tool. Proceedings of the Distributed Multimedia Systems.

[29] Mohammadi N., Wang J., Cao Y., Setareh M. (2013). SMATS: Sketch-based Modeling and Analysis of Truss Systems. ARCC Conf. Repos..

[30] Murugappan S., Piya C., Yang M.C., Ramani K. (2017). FEAsy: A Sketch-Based Tool for Finite Element Analysis. J. Comput. Inf. Sci. Eng..

[31] Alamgir R.M., Shuvro A.A., Al Mushabbir M., Raiyan M.A., Rani N.J., Rahman M.M., Kabir M.H., Ahmed S. (2022). Performance Analysis of YOLO-based Architectures for Vehicle Detection from Traffic Images in Bangladesh. Proceedings of the 2022 25th International Conference on Computer and Information Technology (ICCIT).

[32] Nazir A., Wani M.A. You Only Look Once—Object Detection Models: A Review. Proceedings of the 2023 10th International Conference on Computing for Sustainable Global Development (INDIACom).

[33] Terven J., Córdova-Esparza D.M., Romero-González J.A. (2023). A Comprehensive Review of YOLO Architectures in Computer Vision: From YOLOv1 to YOLOv8 and YOLO-NAS. Mach. Learn. Knowl. Extr..

[34] Sánchez Hernández S., Romero H., Morales A. (2020). A review: Comparison of performance metrics of pretrained models for object detection using the TensorFlow framework. IOP Conf. Ser. Mater. Sci. Eng..

[35] LeNail A. (2019). NN-SVG: Publication-Ready Neural Network Architecture Schematics. J. Open Source Softw..

[36] Deng L. (2012). The MNIST Database of Handwritten Digit Images for Machine Learning Research [Best of the Web]. IEEE Signal Process. Mag..

[37] Zhou L., Zhao J., Li J., Yuan L., Feng J. (2018). Object Relation Detection Based on One-shot Learning. arXiv.

[38] Abadi M., Agarwal A., Barham P., Brevdo E., Chen Z., Citro C., Corrado G.S., Davis A., Dean J., Devin M. (2016). TensorFlow: Large-Scale Machine Learning on Heterogeneous Distributed Systems. arXiv.

[39] Peduzzi P., Concato J., Kemper E., Holford T.R., Feinstein A.R. (1996). A simulation study of the number of events per variable in logistic regression analysis. J. Clin. Epidemiol..

[40] Mckenna F., Scott M., Fenves G. (2010). Nonlinear Finite-Element Analysis Software Architecture Using Object Composition. J. Comput. Civ. Eng..

